# Posterior tibial neurostimulation for faecal incontinence in community-dwelling older adults: long-term outcomes and predictors of response

**DOI:** 10.1007/s10151-026-03379-5

**Published:** 2026-06-22

**Authors:** M. Bosch-Ramírez, L. Sánchez-Guillén, M. J. Alcaide, M. Serrano-Navidad, F. López-Rodríguez-Arias, A. Sánchez-Romero, A. Muñoz-Duyos, A. Arroyo

**Affiliations:** 1 Colorectal Unit, Department of General Surgery, Elche University Hospital, FISABIO, Hospital General Universitario de Elche, Camino de La Almazara 11, 03203 Elche, Alicante Spain; 2 Department of General Surgery, Moisès Broggi Hospital, Consorci Sanitari Integral, Sant Joan Despí, Barcelona Spain

**Keywords:** Faecal incontinence, PTNS, Geriatric, Elderly, Old patients, Tibial nerve stimulation

## Abstract

**Background:**

Faecal incontinence (FI) increases up to 50% with age in institutionalized older individuals, and treatment options have been scarcely studied for older people. Posterior tibial nerve stimulation (PTNS) is a minimally invasive second-line treatment available. This research aims to assess long-term clinical outcomes of PTNS using the Wexner score in community-dwelling patients aged > 65 years, and its impact on quality of life (QoL).

**Methods:**

A prospective cohort study with 61 patients (median age 71 years; 79% women) was conducted. PTNS was administered in three phases over 12 months, with follow-ups (FUs) at 3, 6, 12 and 36 months. Optimal responders (ORs) were defined as achieving a > 50% reduction in Wexner score compared with baseline. Partial responders that presented a 25–50% reduction in Wexner score were also considered as potential long-term ORs.

**Results:**

At the end of treatment, 64% of patients were OR, with sustained improvement in 77% of them at 36 months. Wexner score significantly decreased throughout FUs, from median ten to four (*p* < 0.001). Faecal incontinence quality of life questionnaires (FIQLs) showed limited improvement in depression domain at 6- and 36-month FUs. Faecal urgency improved in a logistic regression analysis (*p* < 0.01). Multivariable logistic regression identified increasing age as independently associated with clinical response (*p* = 0.04).

**Conclusions:**

PTNS was associated with improvement in incontinence severity scores and faecal urgency in selected community-dwelling older adults, although reductions in FI episode frequency and quality of life measures were limited. These findings suggest that PTNS may have a selective role within individualized management strategies, particularly in urgency-predominant symptoms.

**Trial registration number:**

NCT05016453, retrospectively registered in 2021.

## Introduction

Faecal incontinence (FI) is defined as the incapacity to appropriately control bowel movements, resulting in uncontrolled or involuntary leakage of faeces and/or flatus for at least 3 months [1]. The prevalence of FI is estimated to increase progressively with age and institutionalization, reaching up to 50% [2].

FI leads to a high direct and indirect economic burden to the healthcare system and it is an important cause of institutionalization of older people, especially when associated with urinary incontinence (UI) [3, 4].

First-line treatment consists of symptom management by offering toileting advice, reviewing chronic medication, dietary adjustment, antidiarrheal medication and behavioural therapy with biofeedback or pelvic floor rehabilitation [5]. This is successful in 70% of patients; however, for patients that fail to achieve clinical improvement, other therapies are available [6].

Sacral nerve stimulation (SNS) represents the preferred second-line treatment by main European colorectal societies [7–9]. Although incompletely understood, its mechanism relies on providing chronic low-amplitude electrical stimulation of the S3 root, with a success rate of 54%–89% [10].

Side effects include wound infection and pain associated with battery migration [11]. Permanent treatment requires two surgical procedures, with high associated costs [12] and regular follow-up and battery changes, the latter leading to consecutive surgeries [13].

Sharing a similar mechanism of action is posterior tibial nerve stimulation (PTNS), which retrogradely stimulates the same sacral roots as SNS, but from peripheral sites in the lower limb, representing a less invasive and cheaper alternative compared with SNS, with more modest efficacy.

Efficacy of PTNS has been reported between 52% and 82%, being more effective for mild FI. However, evidence is based on small observational studies [14] and four randomized clinical trials (RCT) comparing PTNS with sham stimulation [15], reporting modest results in the short-term, and none of them reporting long-term results.

SNS has been studied in cognitively intact older adults, being effective for UI, but it has been scarcely studied for FI [16]. Similarly, PTNS has only been studied for UI in the older population within the electric tibial nerve stimulation to reduce incontinence in care homes (ELECTRIC) RCT [17] but has not been studied for treating FI.

PTNS has been proposed as a second-line non-surgical treatment in the latest European Guidelines [8], prior to considering surgical interventions. This guideline also highlights that surgical options are less preferable for nursing home patients, but no recommendation could be made about community-dwelling patients, owing to the lack of evidence.

The aims of this study were to evaluate long-term outcomes of PTNS (36 months) in elderly community-dwelling patients (> 65 years old) suffering from FI refractory to conservative treatment in order to describe outcomes for this specific population in the long-term. Secondary aims were assessing QoL of patients in terms of FIQLs and variations in faecal urgency.

## Methods

This is a prospective interventional study with a pre-specified sub-analysis of patients aged > 65 years from a previously published cohort to assess PTNS outcomes in elderly patients with faecal incontinence. Following ethics approval (PI 66/2020), all consecutive patients treated with PTNS between 2010 and 2019 at Elche University Hospital were prospectively followed and invited to participate. The inclusion criteria comprised age > 65 years old, refractory to conservative treatment and including patients with mild-to-severe FI. Exclusion criteria were major psychological or psychiatric comorbidity, lower limb affections that would prevent from correct needle placement (i.e., diabetic ulcers, chronic wounds, extensive limb oedema) and anatomical sphincter defects requiring surgical management. Aetiological classification was heterogeneous and was not sufficiently robust to allow reliable sub-group analysis by underlying mechanism of faecal incontinence.

At the beginning of the study, the patients’ medical history with current medication prescription, physical exam, anal ultrasonography and manometric data were collected. Functional independence was assessed using the Barthel Index [18] and cognitive status by Mini-Mental State Examination (MMSE) [19]. We recorded the onset of symptoms, type of incontinence, 21-day bowel habit diary [20] and number of depositions per day.

The main variable in our study was Wexner Score (or Cleveland Clinic Florida Faecal Incontinence Score) [21], which is a validated severity score that combines type of incontinence and frequency of FI episodes.

Quality of life (QoL) was assessed at baseline and at each follow-up by faecal incontinence quality of life questionnaire (FIQLs) [22] which is a disease-specific test for FI and measures quality of life in four domains (lifestyle, coping/behaviour, depression and embarrassment) on a scale of 1–4. Ability to delay defecation (faecal urgency) was also registered. The Likert scale [23] was used to evaluate patients’ perception of clinical improvement at the end of the study.

Manometric measurement was also performed at 6- and 12-month follow-ups, recording maximal resting pressure (MRP) and maximal squeeze pressure (MSP).

**Fig. 1:**
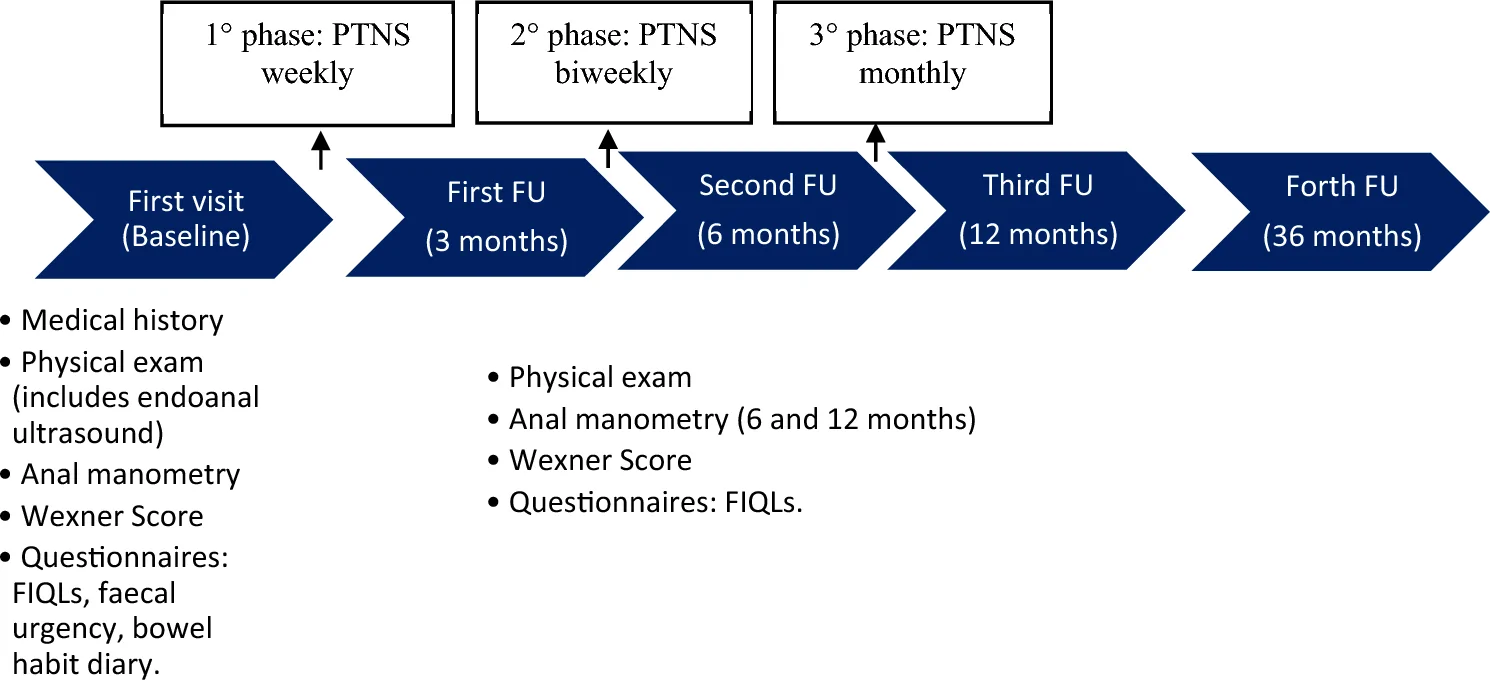
PTNS study flowchart. *FU* follow-up, *FIQLs* faecal incontinence quality of life test

Clinical follow-up was assessed at 3, 6, 12 and 36 months, as detailed in Fig. 1.

### PTNS protocol (Fig. 1)

PTNS sessions were performed in the out-patient clinic and these consistently lasted 30 min. Frequency of these sessions changed depending on the phase of treatment.

The first phase included weekly sessions for 3 months. The second phase led to biweekly sessions for 3 more months and lastly, the third phase consisted of monthly sessions for a duration of 6 months.

Assessment of the efficacy of PTNS was performed after every phase of treatment (3, 6 and 12 months) and at the end of the study (36 months).

PTNS was performed by members of the Colorectal Unit, using Urgent PC stimulator device (Uroplasty, Minnetonka, MN, USA) [24].

According to our previous publication [25] patients were classified as optimal responders (OR) when they experienced a reduction of Wexner score > 50% compared with baseline; partial responders (PR) when reduction was 25–50%; and insufficient responders (IR) when reduction on Wexner score was < 25%. Optimal and partial responders passed on to successive phases of treatment, while insufficient responders were offered other therapeutic options.

### Statistical analysis

Data analysis was performed using SAS software (version 9.4, SAS Institute Inc., Cary, NC, USA). The quantitative results were expressed as the mean (median for the non-normally distributed data), standard deviation (SD), minimum, maximum and quartiles. The categorical variables were expressed as the number of cases and percentages.

Associations between the quantitative and normally distributed variables were analysed using Student’s *t*-test, and Wilcoxon was performed to evaluate non-normal variables. Categorical variables were analysed using the *χ*^2^ test.

Logistic regression and multivariate analysis were performed to estimate independent risk factors for optimal response to treatment as an exploratory analysis for potential predictors of response. Results are presented as odds ratios (OR) with 95% confidence intervals (CI).

The statistical significance was set at *p* < 0.05. No correction for multiple comparisons was applied; therefore, secondary analyses should be interpreted as exploratory. Artificial intelligence tools were used to support clarity and structure.

All conceptual content, interpretation of data and final editorial decisions were made entirely by the authors.

## Results

A total of 61 community-dwelling patients (48 women, 78.7%), with a median age of 71 years (65–82), underwent PTNS for FI refractory to conservative treatment (Table 1). The median functional status by Barthel Index was 75 (45–90) and the median MMSE score was 26 (22–28). Most relevant comorbidities were concomitant UI in 31 patients (50.8%), type 2 diabetes mellitus in 21 patients (34.4%) and previous diagnosis of dementia in 14 patients (23%). Among female patients, 98% had previous obstetric history, episiotomy being the most frequent (48%), more than three vaginal deliveries (37.5%) and vaginal tear (35.4%). Anorectal ultrasound revealed no significant sphincteric abnormalities in 51% of cases.

**Table 1:** Demographic, comorbidities and anorectal characteristics

Demographics	
Age	71 (65–82)
Sex	
Women	48 (78.7%)
Men	13 (21.3%)
Barthel Index	75 (40–90)
MMSE Score	26 (22–28)
Comorbidities	
Type 2 diabetes mellitus	21 (34.4%)
Urinary incontinence	31 (51%)
Dementia	14 (23%)
Previous stroke	2 (3.28%)
Radicular disease	8 (13%)
Gastrointestinal disorders	
Irritable bowel syndrome (IBS)	1 (1.6%)
Crohn’s disease	1 (1.6%)
Ulcerative colitis	1 (1.6%)
Previous oncologic disease	
Colorectal	5 (8,2%)
Prostatic	2 (3.3%)
Previous surgery	
Complex pelvic surgery	15 (24.6%)
Sphincteroplasty	1 (1.6%)
Hysterectomy	12 (25%)
Anorectal previous comorbidity	
Rectocele	4 (6.5%)
Cystocele	3 (4.9%)
Haemorrhoids	6 (9.8%)
Fistula	1 (1.6%)
Fissure	7 (11.5%)
Rectal prolapse	3 (4.9%)
Fibromyalgia	3 (4.9%)
Obstetric past history	
Vaginal tear	17 (35.4%)
Episiotomy	23 (48%)
Delivery with shoulder dystocia	9 (18.7%)
Twin delivery	1 (2%)
Two vaginal deliveries	9 (18.7%)
Three vaginal deliveries	8 (16.7%)
> three vaginal deliveries	18 (37.5%)
Sphincter morphology on Ultrasound	
Intact sphincters	25 (51%)
< 30 lesion	10 (20.4%)
30–90° lesion	14 (28.5%)

*MMSE Score* Mini-Mental State Examination Score

Most patients suffered from mixed FI (60%), 36% had urge FI and only 4% presented passive FI. Incontinence episodes per day at baseline were as follows: 1 episode per day (21%), 2–3 episodes per day (58%) and 4–6 episodes per day (20,7%).

Time of consultation was 1–5 years since the start of symptoms in the majority of cases (41%) and 67.8% showed severe faecal urgency, not being able to delay defecation longer than a minute, and no longer than 5 min in 22.03% of patients.

Baseline mean manometry measures were 25 mmHg MRP (normal value 50–90 mmHg) and 43 mmHg MSP (normal value 120–350 mmHg).

**Fig. 2:**
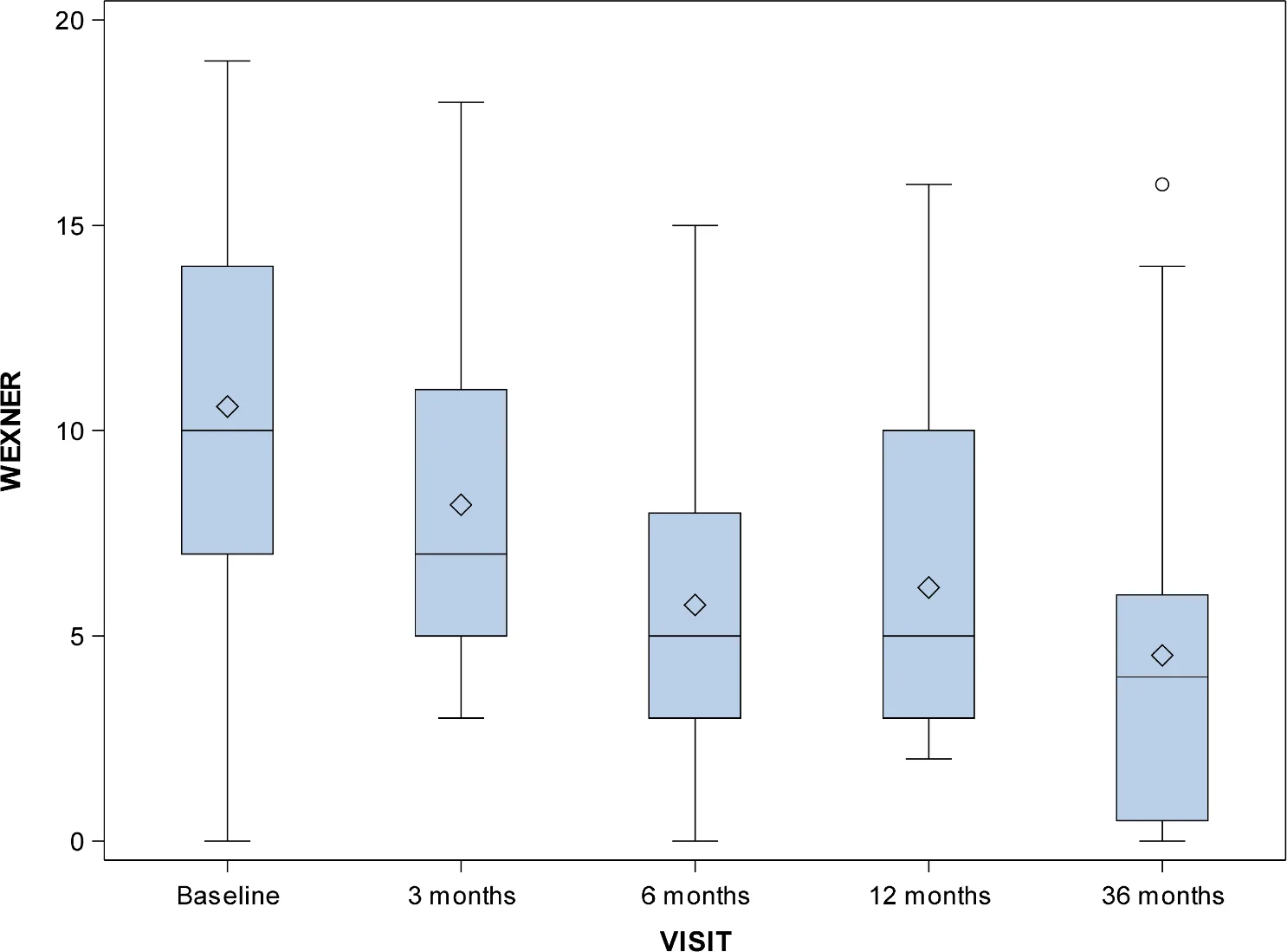
Wexner score punctuation at baseline and follow-up

The median baseline Wexner score was 10.5 (moderate). A statistically significant reduction was observed at all follow-up points (Fig. 2). By the 36-month evaluation, Wexner score showed a sustained decrease with significant differences between baseline and all follow-ups, as well as between the 3-month and both 6-month and 36-month evaluations towards mild severity (*p* < 0.05) (Table 2).

**Table 2:** Post hoc comparisons in Wexner score

Visit	Visit	Estimate	Standard error	*t*-Value	Adj lower	Adj upper	Adj *P*
**Baseline**	**3 months**	**2.7183**	**0.5698**	**4.77**	**1.1418**	**4.2949**	** < 0.0001**
**Baseline**	**6 months**	**4.6066**	**0.5879**	**7.84**	**2.9797**	**6.2334**	** < 0.0001**
**Baseline**	**12 months**	**4.6484**	**1.0105**	**0.60**	**1.8524**	**7.4444**	** < 0.0001**
**Baseline**	**36 months**	**5.1556**	**0.6183**	**8.34**	**3.4446**	**6.8665**	** < 0.0001**
**3 months**	**6 months**	**1.8882**	**0.6286**	**3.00**	**0.1489**	**3.6275**	**0.0261**
3 months	12 months	1.9300	1.0357	1.86	0.9357	−4.7957	0.3423
**3 months**	**36 months**	**2.4372**	**0.6618**	**3.68**	**0.6060**	**4.2685**	**0.0031**
6 months	12 months	0.04181	1.0530	0.04	−2.8719	2.9556	1.0000
6 months	36 months	0.5490	0.6696	0.82	1.3039	−2.4019	0.9241
12 months	36 months	0.5072	1.0444	0.49	−2.3828	3.3972	0.9886

Bold rows highlight comparisons reaching statistical significance versus baseline (*P*< 0.0001).

A decrease in the number of incontinence episodes per day was also seen at the end of the study (36-months), with no statistical difference during the study period and with 24% of patients presenting 1 FI episode per day, 57% patients with 2–3 FI episodes per day and 19% with 4–6 FI episodes per day.

**Fig. 3:**
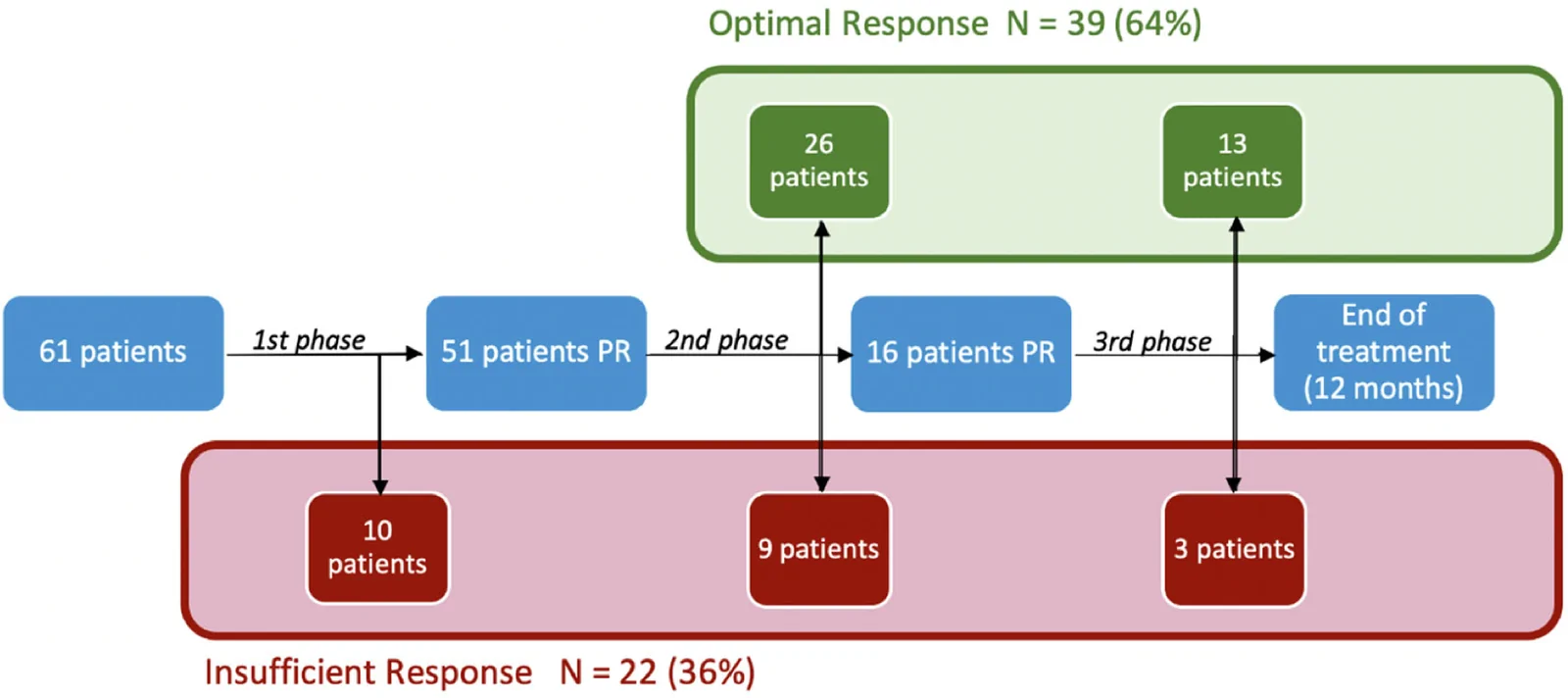
PTNS protocol flowchart. *OR* optimal response, *PR* partial response, *IR* insufficient response

At the end of the 12-month treatment, 39 patients (64%) achieved OR (Fig. 3). No ORs were observed after the first phase; however, 51 patients (83.6%) achieved PR and progressed on to the second phase. After the second phase, 26 patients (42.6%) converted from PR to OR, while 16 patients remained as PR and 9 discontinued treatment owing to insufficient response (IR). During the third phase, an additional 13 patients (21.3%) converted from PR to OR. All patients that obtained OR on previous phases of treatment continued to receive PTNS sessions for up to 12 months.

At the 36-month evaluation of patients previously exhibiting OR, 30 patients (77%) still showed OR after 2 years without any further neuromodulation treatment apart from conservative measures, maintained all along. Therefore, the global response to PTNS at the end of treatment was 64% (39 patients), descending to 49.2% (30 patients) at the end of the study (36-month follow-up).

No adverse events were observed secondary to PTNS. Two patients discontinued treatment owing to bimalleolar oedema secondary to cardiac insufficiency and one patient died owing to respiratory comorbidity, both during the second phase (considered IR).

**Fig. 4:**
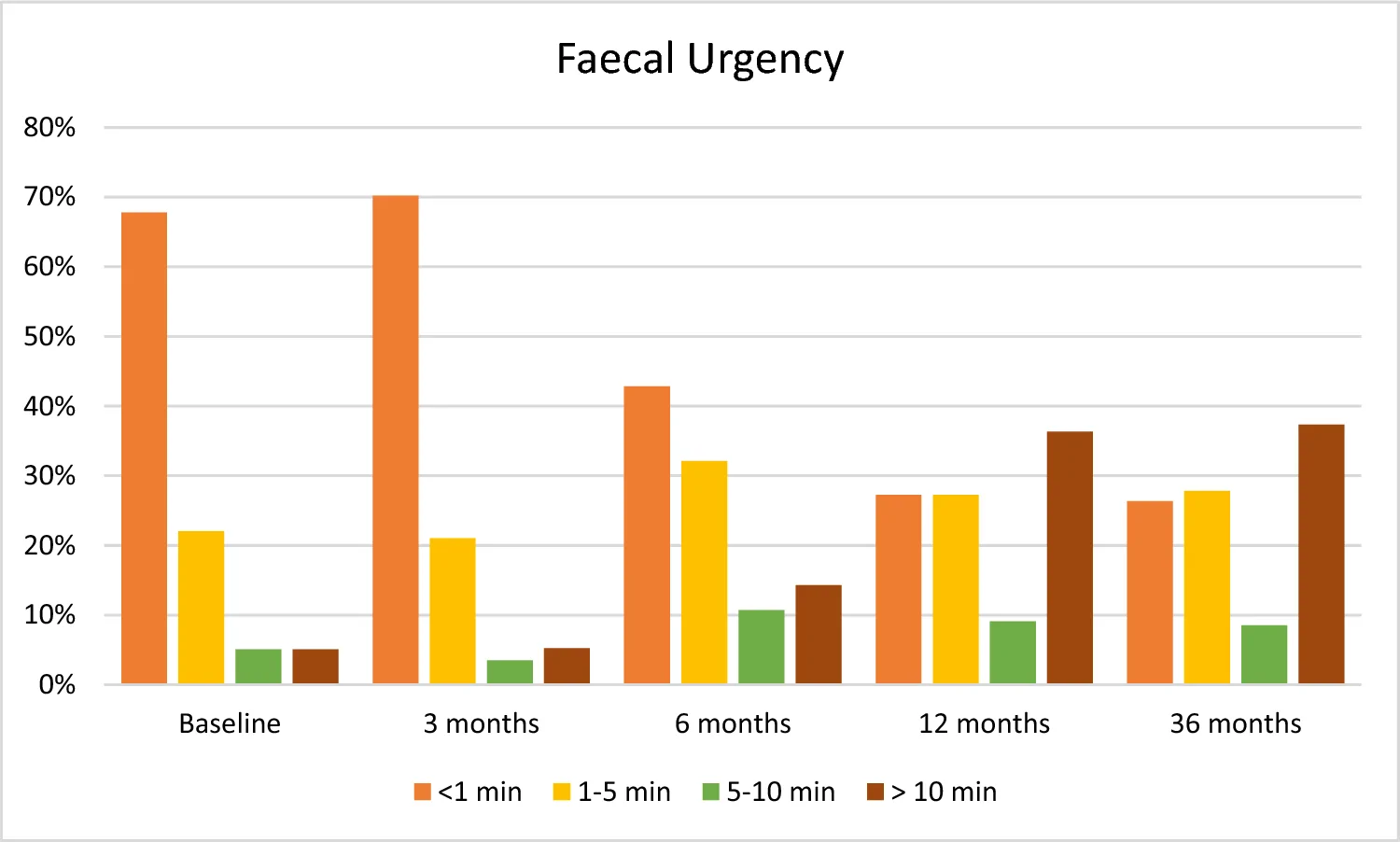
Faecal urgency

Faecal urgency showed a general trend towards improvement, close to statistical significance (*p* = 0.059), with 67.8% of patients unable to delay defecation > 1 min at baseline, reducing to 27.2% at the end of treatment. By contrast, only 5.1% of patients could delay defecation > 10 min at baseline, which ascended to 36.3% at the final evaluation (Fig. 4), both maintained in the long-term.

Logistic regression for faecal urgency showed statistically significant differences attaining reduction of number of patients unable to delay defecation up to 1 min (*p* = 0.0046) after PTNS. These differences were found in a post hoc analysis to happen between baseline with 6-month follow-up, OR 4.902 CI: 95% [1.099–21.739] (*p* = 0.0327) and between 3-month and 6-month follow-up, OR 5.5 CI: 95% [1.217–25.59] (*p* = 0.0205).

Also, an increase in patients able to delay defecation more than 10 min (*p* = 0.0138) was found, when comparing baseline with 12-month follow-up, OR 0.047 CI: 95% [0.003–0.692] (*p* = 0.0192) and 3-month with 12-month follow-up, OR 0.048 CI: 95% [0.003–0.711] (*p* = 0.0207) (Table 3).

**Table 3:** Logistic regression for faecal urgency in geriatric patients treated with PTNS

Faecal urgency < 1 min					Faecal urgency > 10 min		
Visit	Visit	OR	CI 95%	*p*	OR	CI 95%	*p*
Baseline	3 months	0.877	0.252–3.048	0.9928	0.976	0.90–10.526	1.0000
Baseline	6 months	**4.902**	**1.099–21.739**	**0.0327**	0.301	0.028–3.246	0.5526
Baseline	12 months	7.518	0.757–7.518	0.1055	**0.047**	**0.003–0.692**	**0.0192**
3 months	6 months	**5.580**	**1.217**–**25.598**	**0.0205**	0.309	0.029–3.339	0.5707
3 months	12 months	8.547	0.851–83.33	0.0778	**0.048**	**0.003–0.711**	**0.0207**
6 months	12 months	1.538	0.136–17.241	0.9664	0.158	0.011–2.212	0.2667

Bold values denote significant odds ratios and their corresponding *p*-values

Manometric evaluations showed no statistically significant changes throughout the study (MRP: *p* = 0.28; MSP: *p* = 0.91).

The FIQLs showed no significant global differences (*p* = 0.1675). Significant improvements were observed in the depression domain at both 6-month (*p* = 0.0467) and 36-month follow-ups (*p* = 0.0407). At the end of the study, 75.7% of patients perceived relevant clinical improvement through the Likert scale.

Logistic regression was used for further analysis to determine predictors of clinical improvement after PTNS. Increasing age was the only variable independently associated with clinical improvement (*p* = 0.0444), with a median age of 68 years old in the group with no clinical improvement versus 72.5 years old in the group with clinical improvement. Barthel, gender, urinary incontinence and dementia did not predict clinical response (*p* > 0.05). These findings continued to be significant in a multivariate analysis [OR 1.311 (CI: 95% (1.007–1.7)] *p* = 0.0444 (Table 4).

**Table 4:** Results of multivariable logistic regression analysis on the outcome of clinical improvement in faecal incontinence in older patients treated with PTNS

Effect	Wald chi-square	Pr > ChiSq	OR (CI 95%)
Barthel	0.1718	0.6785	
**Age**	**4.0407**	**0.0444**	**1.311 (1.007–1.708)**
Sex	0.1353	0.7130	
Urinary incontinence	0.0220	0.8820	
Dementia	0.8150	0.3666	

Bold row indicates the only variable independently associated with clinical improvement (Age, *p* = 0.0444)

Owing to higher prevalence of FI in female patients and 98% of our female undergoing past obstetric history, post hoc analyses were performed in order to predict prognostic factors for response, with no statistically significant findings (*p* > 0.05).

## Discussion

To our knowledge, this is the first study to evaluate long-term outcomes of PTNS exclusively in patients aged > 65 years with FI refractory to conservative treatment. Our findings suggest that PTNS was associated with clinically meaningful improvement and demonstrated a favourable safety profile in community-dwelling elderly patients, with a substantial proportion of patients maintaining clinical improvement at the final evaluation.

The primary outcome of this study was reduction in Wexner score, which showed a progressive and statistically significant decrease from moderate FI to mild affliction at all follow-up intervals. At the end of treatment (12 months), 64% of patients presented OR and this response was maintained in 77% of the responder patients at the 36-month follow-up, although long-term durability of PTNS beyond this period remains uncertain.

Although validated severity scoring instruments for FI such as Wexner [21], Vaizey (St. Marks) [26] and Faecal Incontinence Severity Index [27] are widely used, episodes of FI per week registered in a bowel diary has classically been the main tool used in literature to measure response to treatment, which hinders comparison of results between groups [28]. However, 64% of optimal response in Wexner Score at the end of treatment suggests that older patients with FI can achieve comparable benefits to those reported in previous broader adult PTNS cohorts. In contrast to Wexner improvement, the number of FI episodes per day did not decrease significantly during follow-up. This may be explained by the fact that PTNS better improved FI with urgency symptoms but did not significantly decrease number of incontinence episodes. Ability to delay defecation improved in a logistic regression, with a reduction in number of patients unable to delay defecation up to 1 min (*p* = 0.0046) and an increase in patients able to delay defecation over 10 min (*p* = 0.0138); which suggests that patients with mixed or urgency FI could better benefit from PTNS compared with passive FI.

PNTS efficacy has been studied in four RCTs. George et al. first randomized 30 patients to transcutaneous-PTNS (T-PTNS), percutaneous-PTNS (P-PTNS) and sham stimulation [29] twice weekly for 6 weeks. Results showed benefit of PTNS over sham stimulation (*p* = 0.035), favouring percutaneous route. Control of faecal incontinence using distal neuromodulation (CONFIDeNT) trial [30] was a multicentre, double-blinded, pragmatic RCT that included 227 patients with FI allocated to PTNS versus sham stimulation. Although initial results for 12-week treatment did not show statistical differences between groups, a post hoc analysis [14] excluding patients with obstructive disorder syndrome did show clinical benefit of PTNS over sham stimulation (48.9% versus 18.2% response, *p* = 0.002; multivariable OR: 4.71; CI 1.71–12.93; *p* = 0.003).

Van der Wilt et al. performed a single-blinded RCT, randomizing 59 patients to either PTNS or sham stimulation for 9 weeks [31] and showed small advantage of PTNS over sham stimulation (*p* = 0.028). Lastly, the neuromodulation for accidental bowel leakage (NOTABLe) trial [32] focused on female population and randomized patients to PTNS and sham stimulation for 12 weeks and differences were not statistically significant.

All these RCTs share the same duration of treatment (12–15 weeks), according to the previous standard of treatment based on overactive bladder physiology. However, as our team [25] shared, initial rates of OR (after 12 weeks) can be limited, but these findings improved up to 64% after three phases of treatment. This could only be possible owing to the perseverance of PTNS in patients that showed initial PR (decrease between 25% and 50% of Wexner).

For elderly patients, long-term efficacy (36 months) decreases progressively over time, with a 36-month efficacy of 49.2%, similar to our previous long-term results in the general population. This might leave room to study efficacy of top-up or maintenance sessions in elderly patients, as previously evaluated for global population [33].

Only one study [20] has evaluated sacral nerve stimulation (SNS) exclusively in older patients with faecal incontinence. George et al. (*n* = 23) reported significant long-term improvements at 44 months in weekly episodes, St Mark’s score, faecal urgency and deferral time. The permanent nature of SNS may explain its sustained efficacy and supports exploring PTNS maintenance sessions in older adults. Neither their study nor ours showed significant changes in resting or squeeze pressures, consistent with neuromodulation acting mainly on sensory afferent pathways rather than restoring structural sphincter function [34].

Whereas SNS currently retains the stronger evidence base for long-term continence outcomes, PTNS may be considered as a complementary non-surgical option in selected older adults, particularly in urgency-predominant symptoms or in patients less suitable for invasive procedures. However, PTNS requires repeated treatment sessions over time; therefore, accessibility to medical care and potential risks of repeated subcutaneous puncture might represent a relevant burden for elderly patients, although the experience of this team regarding side effects and adherence to treatment over time has been positive.

FIQL scores did not show overall significant improvement; only the depression domain improved at isolated time points, so results should be interpreted cautiously. Recent randomised trials [30, 32] found no differences between PTNS and sham for FIQL or 36-item short form survey(SF-36) after 9–12 weeks. Earlier small cohorts reported QoL benefits, but FIQL complexity may limit completion in older patients. QoL perception is multifactorial; notably, 75.68% of participants rated outcomes as satisfactory on a simple Likert scale, suggesting perceived benefit despite limited changes in standardised QoL instruments. These findings suggest that the inclusion of a generic quality of life instrument, such as SF-36 or EQ-5D, may be useful in future studies to better capture patient-perceived benefit and improve comparability across interventions.

The dissociation between improvements in composite severity scores and the limited changes observed in episode frequency and QoL measures suggests that statistical improvement should not necessarily be interpreted as complete restoration of continence.

Interestingly, older age emerged as a positive predictor of long-term optimal response. However, this finding should be interpreted with care. The relatively small sample size may increase the risk of a small-sample effect and potential model overfitting in multivariable analysis. Furthermore, the biological plausibility of older age conferring a better neuromodulatory response remains unclear. These results should therefore be considered hypothesis-generating and require confirmation in larger, adequately powered studies. Importantly, because our cohort consisted exclusively of community-dwelling older adults with relatively preserved functional and cognitive status, these findings cannot be extrapolated to frail elderly individuals.

Several limitations of this study should be acknowledged. The lack of standardised outcome measures and protocols hinders international comparisons. The lack of a younger comparison group restricts assessment of whether age itself has a meaningful impact on the response to neuromodulation. Community-dwelling, relatively fit participants limit generalizability; therefore, nursing-home and hospitalized patients would benefit from specific assessment, as Booth et al. did in care homes for UI [17]. The sample size, while adequate for statistical analysis, limits the ability to perform subgroup analyses to explore additional predictors of response. Lastly, the absence of a control group is also a significant limitation. Given that no correction for multiple comparisons was applied, secondary findings should be interpreted as exploratory.

The observational design of the study precludes establishing a causal relationship between PTNS and clinical improvement. Further research is needed to confirm these findings in larger, multicentre RCT.

## Conclusions

PTNS may represent a complementary non-surgical option for selected community-dwelling older adults, particularly in urgency-predominant symptoms, although its impact on leakage frequency and quality of life appears modest. Further randomized studies are needed to better define its role within the therapeutic algorithm, including community-dwelling and nursing-home patients.

## Data Availability

No datasets were generated or analysed during the current study.

## References

[1] Rao SS, Bharucha AE, Chiarioni G, Felt-Bersma R, Knowles C et al (2016) Anorectal disorders. Gastroenterology 150(6):1430–1442. 10.1053/j.gastro.2016.02.009 PMC5035713 27144630

[2] Ihnát P, Kozáková R et al (2016) Fecal incontinence among nursing home residents: is it still a problem? Arch Gerontol Geriatr 65:79–84. 10.1016/j.archger.2016.03.012 27010346

[3] Borrie MJ, Davidson HA (1992) Incontinence in institutions: costs and contributing factors. CMAJ Canad Med Assoc J 147(3):322–8 (**PMID: 1643598**) 1643598 PMC1336202

[4] Carvalho N, Fustinoni S, Abolhassani N et al (2020) Impact of urine and mixed incontinence on long-term care preference: a vignette-survey study of community-dwelling older adults. BMC Geriatr 20(1):69. 10.1186/s12877-020-1439-x 32070294 PMC7029586

[5] Musa MK, Saga S, Blekken LE et al (2019) The prevalence, incidence, and correlates of fecal incontinence among older people residing in care homes: a systematic review. J Am Med Dir Assoc 20(8):956-962.e8. 10.1016/j.jamda.2019.03.033 31129021

[6] Wald A, Bharucha AE, Cosman BC, Whitehead WE (2014) ACG clinical guideline: management of benign anorectal disorders. Am J Gastroenterol 109(8):1141–1157. 10.1038/ajg.2014.190 25022811

[7] Maeda Y, O’Connell PR, Lehur PA, Matzel KE, Laurberg S (2015) European SNS bowel study group. Sacral nerve stimulation for faecal incontinence and constipation: a European consensus statement. Colorectal Dis 17:O74-87. 10.1111/codi.12905 25603960

[8] Assmann SL, Keszthelyi D et al (2022) Guideline for the diagnosis and treatment of faecal incontinence—a UEG/ ESCP/ESNM/ESPCG collaboration. Unit Eur Gastroenterol J 10(3):251–286. 10.1002/ueg2.12213 PMC9004250 35303758

[9] Cerdán Miguel J, Arroyo Sebastián A, Codina Cazador A, de Juan FD et al (2024) Baiona’s consensus statement for fecal incontinence Spanish association of coloproctology. Cir Esp. 102(3):158–173. 10.1016/j.cireng.2023.07.008 38242231

[10] Altomare DF, Giuratrabocchetta S, Knowles CH, Muñoz Duyos A et al (2015) European SNS outcome study group long-term outcomes of sacral nerve stimulation for faecal incontinence. Br J Surg. 102(4):407–15. 10.1002/bjs.9740 25644687

[11] Thin NN, Horrocks EJ, Hotouras A et al (2013) Systematic review of the clinical effectiveness of neuromodulation in the treatment of faecal incontinence. Br J Surg 100(11):1430–1447. 10.1002/bjs.9226 24037562

[12] Hetzer FH, Bieler A et al (2006) Outcome and cost analysis of sacral nerve stimulation for faecal incontinence. Br J Surg 93(11):1411–1417. 10.1002/bjs.5491 17022014

[13] Thin NN, Taylor SJ, Bremner SA, Emmanuel AV, Hounsome N, Williams NS, Knowles CH, Neuromodulation trial study group (2015) randomized clinical trial of sacral versus percutaneous tibial nerve stimulation in patients with faecal incontinence. Br J Surg 102(4):349–58. 10.1002/bjs.9695 25644291

[14] Horrocks EJ, Chadi SA, Stevens NJ, Wexner SD, Knowles CH (2017) Factors associated with efficacy of percutaneous tibial nerve stimulation for fecal incontinence, based on post-hoc analysis of data from a randomized trial. Clin Gastroenterol Hepatol 15(12):1915-1921.e1912. 10.1016/j.cgh.2017.06.032 28647458

[15] Luo C, Wei D, Pang K, Mei L et al (2024) Is percutaneous tibial nerve stimulation (PTNS) effective for fecal incontinence (FI) in adults compared with sham electrical stimulation? A meta-analysis. Tech Coloproctol 28(1):37. 10.1007/s10151-024-02910-w. (**Erratum. In: (2024) Tech Coloproctol. 9;28(1):52. 10.1007/s10151-024-02924-4**) 38401006

[16] George AT, Kalmar K, Goncalves J, Nicholls RJ, Vaizey CJ (2011) Sacral nerve stimulation in the elderly. Col Dis 14:200–204. 10.1111/j.1463-1318.2011.02568.x 21689281

[17] Booth J, Aucott L, Cotton S et al (2021) Tibial nerve stimulation compared with sham to reduce incontinence in care home residents: ELECTRIC RCT. Health Technol Assess 25(41):1–110. 10.3310/hta25410 34167637 PMC8273680

[18] Stone SP, Ali B, Aurberleek J, Thompsell A, Young A (1994) The Barthel index in clinical practice: use on a rehabilitation ward for elderly people. J Roy Coll Phys Lond 28:491–423 (**PMCID: PMC5401036**) PMC5401036 7807430

[19] Folstein MF, Folstein SE, McHugh PR (1975) “Mini-mental state”. A practical method for grading the cognitive state of patients for the clinician. J Psychiatr Res. 12(3):189–98. 10.1016/0022-3956(75)90026-6 1202204

[20] Muñoz-Duyos A, Lagares-Tena L, Vargas-Pierolas H, Rodón A, Navarro-Luna A (2017) High-resolution circuit for the diagnosis of faecal incontinence. Patient satisfaction. Cir Esp (English Ed) 95(5):276–282. 10.1016/j.ciresp.2017.04.012 28602392

[21] Jorge JM, Wexner SD (1993) Etiology and management of fecal incontinence. Dis Colon Rectum 36(1):77–97. 10.1007/BF02050307 8416784

[22] Rockwood TH, Church JM, Fleshman JW, Kane RL, Mavrantonis C, Thorson AG et al (2000) Fecal incontinence quality of life scale: quality of life instrument for patients with fecal incontinence. Dis Colon Rectum 43(1):9–16. 10.1007/BF02237236 10813117

[23] Jebb AT, Ng V, Tay L (2021) A review of key Likert scale development advances: 1995–2019. Front Psychol 12:637547. 10.3389/fpsyg.2021.637547 34017283 PMC8129175

[24] Hotouras A, Thaha MA, Allison ME et al (2012) Percutaneous tibial nerve stimulation (PTNS) in females with faecal incontinence: the impact of sphincter morphology and rectal sensation on the clinical outcome. Int J Colorectal Dis 27(7):927–930. 10.1007/s00384-011-1405-3 22274577

[25] Bosch-Ramírez M, Sánchez-Guillén L, Alcaide-Quirós MJ, Aguilar-Martínez MM, Bellón-López M, López Delgado A, López-Rodríguez-Arias F, Muñoz-Duyos A, Barber-Valles X, Arroyo A (2023) Long-term efficacy of percutaneous tibial nerve stimulation for faecal incontinence and a new approach for partial responders. Tech Coloproctol 27(6):443–451. 10.1007/s10151-022-02711-z 36222850 PMC10169891

[26] Vaizey CJ, Carapeti E, Cahill JA, Kamm MA (1999) Prospective comparison of faecal incontinence grading systems. Gut 44:77–80. 10.1136/gut.44.1.77 9862829 PMC1760067

[27] Rockwood TH, Church JM, Fleshman JW et al (1999) Patient and surgeon ranking of the severity of symptoms associated with fecal incontinence: the fecal incontinence severity index. Dis Colon Rectum 12:1525–1532. 10.1007/BF02236199 10613469

[28] Vaizey CJ (2014) Faecal incontinence: standardizing outcome measures. Colorectal Dis 16:156–158. 10.1111/codi.12566 24450821

[29] George AT, Kalmar K, Sala S et al (2013) Randomized controlled trial of percutaneous versus transcutaneous posterior tibial nerve stimulation in faecal incontinence. Br J Surg 100(3):330–338. 10.1002/bjs.9000 23300071

[30] Knowles CH, Horrocks EJ, Bremner SA et al (2015) Percutaneous tibial nerve stimulation versus sham electrical stimulation for the treatment of faecal incontinence in adults (CONFIDeNT): a double-blind, multicentre, pragmatic, parallel-group, randomised controlled trial. Lancet 386(10004):1640–1648 26293315 10.1016/S0140-6736(15)60314-2

[31] van der Wilt AA, Giuliani G, Kubis C, van Wunnik BPW et al (2017) Randomized clinical trial of percutaneous tibial nerve stimulation versus sham electrical stimulation in patients with faecal incontinence. Br J Surg 104(9):1167–1176. 10.1002/bjs.10590 28703936

[32] Zyczynski HM, Richter HE, Sung VW et al (2022) Percutaneous tibial nerve stimulation vs sham stimulation for fecal incontinence in women: neuromodulation for accidental bowel leakage randomized clinical trial. Am J Gastroenterol 117(4):654–667. 10.14309/ajg.0000000000001605 35354778 PMC8988447

[33] Hotouras A, Murphy J, Walsh U, Allison M, Curry A, Williams NS et al (2014) Outcome of percutaneous tibial nerve stimulation (PTNS) for fecal incontinence: a prospective cohort study. Ann Surg 259(5):939–943 23979291 10.1097/SLA.0b013e3182a6266c

[34] Fox JC, Fletcher JG, Zinsmeister AR et al (2007) Effect of aging on anorectal and pelvic floor functions in females. Dis Colon Rectum 49(11):1726–35. 10.1007/s10350-006-0657-4. (**Erratum. In: (2007) Dis Colon Rectum. 50(3):404**) 17041752

